# P386: Bacterial contamination of health care provider’s pagers in a tertiary care hospital

**DOI:** 10.1186/2047-2994-2-S1-P386

**Published:** 2013-06-20

**Authors:** N Al Otaibi, A Alothman, K Mohajer, A Albadani, S Aljohani, A Al Mohanna

**Affiliations:** 1 Medicine, King Abdulaziz Medical City, Riyadh, Saudi Arabia

## Introduction

In this era where nosocomial infections became a great threat to all tertiary care hospitals; we need to evaluate all modifiable risk factors for such morbidity. Medical instruments were thoughts to be involved in transmitting nosocomial isolates.

## Methods

Health care providers (HCP) were chosen randomly in our tertiary care center, and had to fill a questionnaire and submit their bleep to be swabbed and serially numbered and sent to our microbiology lab to be cultured for 48 hours.

## Results

120 pagers of 120 health care providers in our hospital were swabbed. The hundred twenty HCPs are classified as physician (84.2%, respiratory therapist (5.0%), patient educator (3%), nurses (7.5%) and clinical pharmacist (0.8%) 23.3% of physicians belong to Pediatric Department, while 19.2% from the Department of Medicine; 15.1% of physicians were from Department of Surgery. According from the questionnaire, 62.5% of the subjects never disinfect their pagers while 5% did disinfect their pagers daily. The microbiologic data showed that all pagers were contaminated by bacteria except two (1.7%). The leading organism was coagulase-negative staphylococcus at 84.2%; 2 pagers were contaminated by fungal elements (1.7%); 1 pager was contaminated by methicillin-resistant staphylococcus aureus (0.8%).

## Conclusion

Pagers as well as other instruments used for patient’s health can be contaminated by bacteria. A protocol supposed to be made to disinfects, pagers, and all HCPs are supposed to be taught this protocol.

## Disclosure of interest

None declared

